# Metal Cations in G-Quadruplex Folding and Stability

**DOI:** 10.3389/fchem.2016.00038

**Published:** 2016-09-09

**Authors:** Debmalya Bhattacharyya, Gayan Mirihana Arachchilage, Soumitra Basu

**Affiliations:** Department of Chemistry and Biochemistry, Kent State UniversityKent, OH, USA

**Keywords:** G-quadruplex, metal ion coordination, RNA, DNA, stability, structure, polymorphism

## Abstract

This review is focused on the structural and physicochemical aspects of metal cation coordination to G-Quadruplexes (GQ) and their effects on GQ stability and conformation. G-quadruplex structures are non-canonical secondary structures formed by both DNA and RNA. G-quadruplexes regulate a wide range of important biochemical processes. Besides the sequence requirements, the coordination of monovalent cations in the GQ is essential for its formation and determines the stability and polymorphism of GQ structures. The nature, location, and dynamics of the cation coordination and their impact on the overall GQ stability are dependent on several factors such as the ionic radii, hydration energy, and the bonding strength to the O6 of guanines. The intracellular monovalent cation concentration and the localized ion concentrations determine the formation of GQs and can potentially dictate their regulatory roles. A wide range of biochemical and biophysical studies on an array of GQ enabling sequences have generated at a minimum the knowledge base that allows us to often predict the stability of GQs in the presence of the physiologically relevant metal ions, however, prediction of conformation of such GQs is still out of the realm.

## Introduction

G-quadruplex (GQ) is a non-canonical nucleic acid structure that is extensively involved in regulation of a number of biological processes. Monovalent metal ions are a central requirement for the formation of GQ structures. This review primarily focuses on the role of monovalent and divalent metal ions toward the stability and conformational heterogeneity of GQ structures.

Since the time Watson and Crick proposed a model of DNA, the canonical DNA structure has largely been thought to exist as a double stranded right-handed helix (Watson and Crick, 1953) which has provided the basis for our understanding of the genetic code. Since then several non-canonical DNA structures have been discovered and are found to regulate various cellular processes which established their importance in the regulation of biological functions (Saini et al., 2013). In 1910, it was demonstrated that high concentrations of guanylic acid (GMP) can form gels in aqueous solution (Bang, 1910). Almost fifty years later, the structure of GQ was revealed based on X-ray fiber diffraction studies, where each guanine residue in G-quartet acts as both acceptor and donor of two hydrogen bonds via Hoogsteen base pairing (Gellert et al., 1962).

## Biological roles of G-quadruplexes

**Figure 1:**
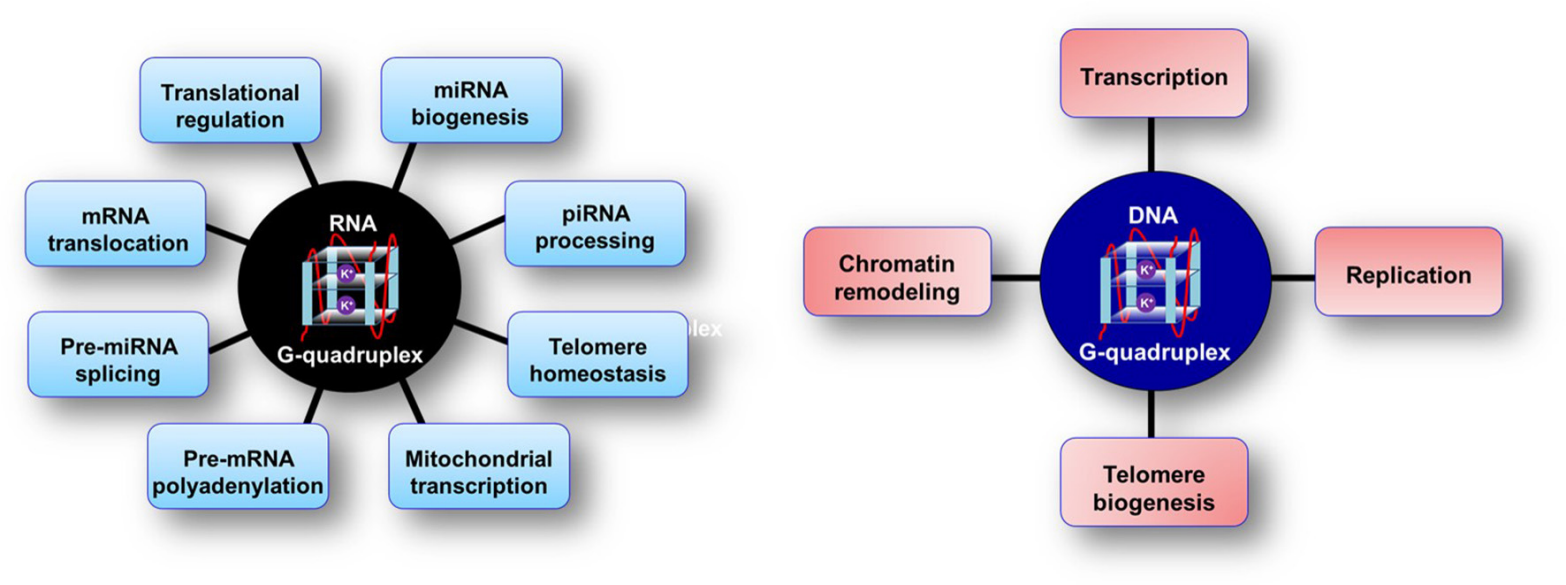
**Cellular processes influenced and modulated by RNA and DNA G-quadruplex structures**.

In the 1980s, studies revealed G-rich repeat sequences in the human telomere were capable of forming GQ structures *in vitro* (Wang and Patel, 1993) and these DNA GQ structures were shown to inhibit the activity of telomerase, an enzyme which is often overexpressed in cancer cells turning them immortal (Zahler et al., 1991). In the post-genomic era, both DNA, and RNA GQs have been shows to play numerous regulatory roles in controlling a myriad of biological processes (Figure 1). Bioinformatics studies have revealed the prevalence of guanine-rich sequences throughout the human genome (Huppert and Balasubramanian, 2005; Todd et al., 2005; Eddy and Maizels, 2008; Yadav et al., 2008; Mani et al., 2009; Neidle, 2009) especially in some of the key growth regulatory genes and oncogenes (Simonsson et al., 1998; Siddiqui-Jain et al., 2002; Sun et al., 2005; Cogoi and Xodo, 2006; Dexheimer et al., 2006). In the human genome, existence of 376,000 DNA putative G-quadruplex forming sequences (PQS) was discovered with significant enrichment of such sequences in the promoter regions (Huppert and Balasubramanian, 2005, 2007). The PQS can be described as a G-rich sequence with at least four stretches of G residues where each stretch is comprised of at least two Gs. A typical PQS is defined as G_≥2_ N_1-7_ G_≥2_ N_1-7_ G_≥2_ N_1-7_ G_≥2_. The number of tiers in the GQ is limited by the number of G residues in the shortest stretch of the contiguous guanines. The regulation of transcription by DNA GQ structures in the promoter regions of clinically significant genes such as C-MYC, BCL-2, C-KIT, K-RAS has been well established (Siddiqui-Jain et al., 2002; De Armond et al., 2005; Cogoi and Xodo, 2006; Dai et al., 2006; Fernando et al., 2006) and can be potential targets for chemotherapeutics (De Cian et al., 2008; Huppert, 2008; Monchaud and Teulade-Fichou, 2008; Balasubramanian and Neidle, 2009; Nielsen and Ulven, 2010; Zhang S. et al., 2014). Several DNA PQS were observed in the immunoglobulin heavy chain switch regions and at mutational hotspots. Therefore, they have been implicated in the maintenance of chromosomal integrity, regulation of replication, transcription and recombination processes (Simonsson, 2001).

In the context of DNA, GQ formation would require unwinding of the two strands, however, there is no structural or physicochemical barrier toward formation of RNA GQ structures (Kim et al., 1991; Cheong and Moore, 1992). Moreover, the GQ formation by RNA can be more facile than their DNA counterparts owing to the absence of a competing complementary strand. Additionally, RNA GQ structures were observed to be more stable than their DNA versions (Cheong and Moore, 1992; Sacca et al., 2005; Kumari et al., 2007). Bioinformatics studies suggested that there are ~3000 5′-UTRs containing at least one RNA PQS (Kumari et al., 2007; Huppert et al., 2008). Several studies established the role of RNA GQs in the regulation of translation. RNA G-quadruplex structures in the 5′-UTR have been shown to repress translation of several clinically important genes such as NRAS, Zic-1, VEGF, TRF2, ERS1, THRA, BCl-2 (Kumari et al., 2007; Arora et al., 2008; Balkwill et al., 2009; Morris and Basu, 2009; Beaudoin and Perreault, 2010; Gomez et al., 2010; Morris et al., 2010; Shahid et al., 2010; Bugaut and Balasubramanian, 2012; Huang et al., 2012; Weng et al., 2012; Agarwala et al., 2013, 2014; Wolfe et al., 2014; Bhattacharyya et al., 2015; Cammas et al., 2015; Kwok et al., 2015). These GQ motifs exhibited a dual mode of regulation wherein several naturally occurring RNA GQs have been shown to have an inhibitory effect on translation, while others were determined to be essential for translation (Bonnal et al., 2003; Morris et al., 2010; Agarwala et al., 2013, 2014). Subsequently, various studies reported the presence of RNA GQs and their functional significance in the coding region, microRNA biogenesis, long non-coding RNAs and telomeric ends (Arora and Suess, 2011; Ji et al., 2011; Jayaraj et al., 2012; Beaudoin and Perreault, 2013; Endoh et al., 2013; Martadinata and Phan, 2013; Bhattacharyya et al., 2014; Mirihana Arachchilage et al., 2015).

## Structure of G-quadruplexes

**Figure 2:**
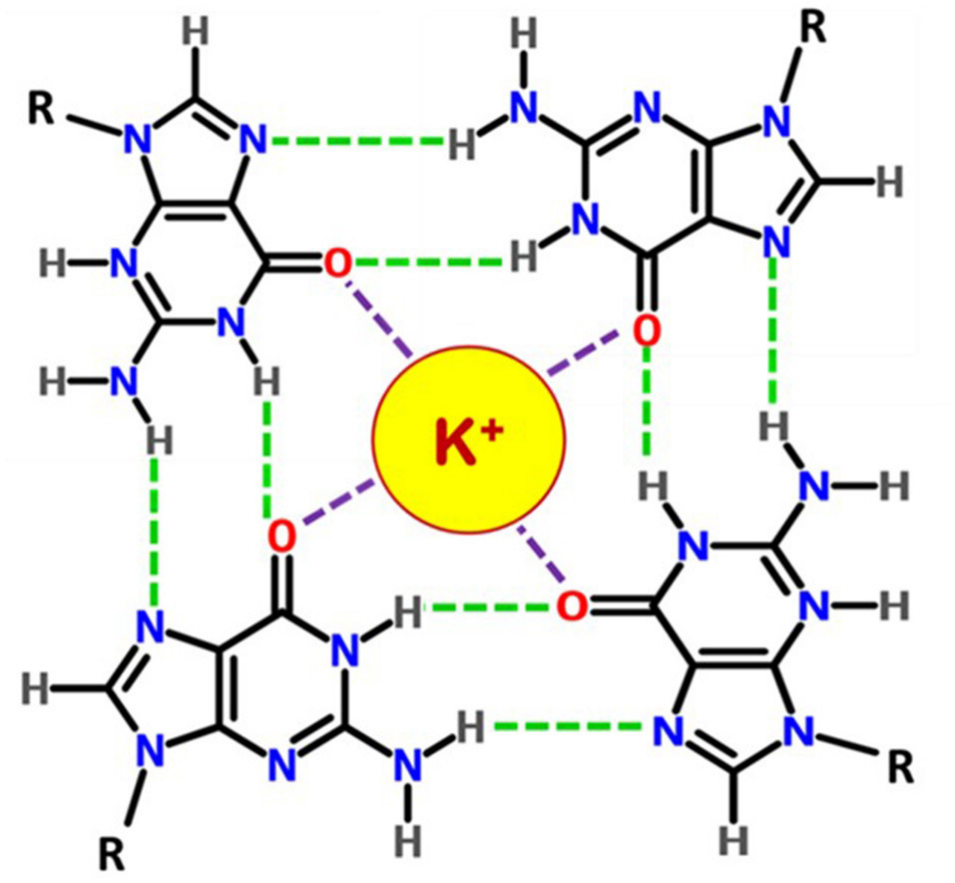
**Chemical structure of a G-quartet**. Four guanosines are hydrogen bonded (green dashes) by Hoogsteen base pairings and the monovalent cation K^+^ interacts with O6 atoms (in red).

**Figure 3:**
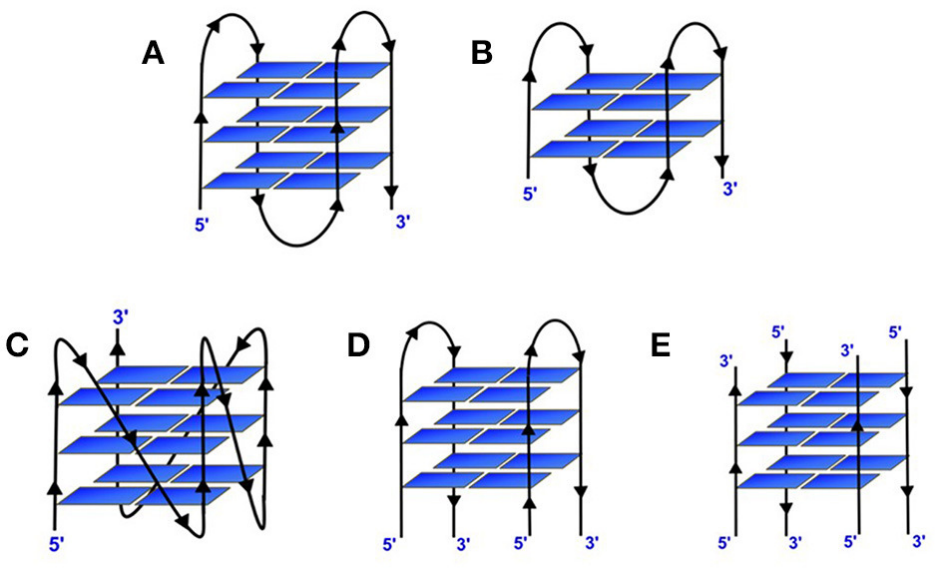
**Different topological variants of G-quadruplexes. (A)** Intramolecular antiparallel 3-tiered GQ; **(B)** Intramolecular antiparallel 2-tiered GQ; **(C)** Intramolecular parallel 3-tiered GQ; **(D)** Bimolecular antiparallel 3-tiered and **(E)** Tetramolecular antiparallel 3-tiered GQ.

G-quadruplex structures are non-canonical four stranded secondary structures found in nucleic acid sequences that are rich in guanine residues. Four guanines can form a square planar tetrad in which each guanine serves as both a hydrogen bond donor and an acceptor. The pairing of the N1 on the first guanine with the O6 on the second guanine along with the pairing of N2 on the first guanine with the N7 on the second guanine results in eight hydrogen bonds per G-tetrad. These structures consist of stacks of G-quartets which are cyclic planar arrangement of four Hoogsteen hydrogen-bonded guanine residues (Figure 2; Smith and Feigon, 1992). Two or more of these G-tetrads stack upon each other to form the GQ structure. The GQ structures exhibit diverse topologies depending on the presence of monovalent cation (K^+^ or Na^+^), conformation of the glycosidic bond (syn or anti); number of molecules of nucleic acid involved in their formation such as, intramolecular/unimolecular (Figures 3A–C), bimolecular (Figure 3D) or tetramolecular (Figure 3E); relative orientation of the strands leading to parallel (Figure 3C) or antiparallel (Figure 3A); the number of stacking G-quartets and the nucleotide sequence (Keniry, 2001; Neidle and Balasubramanian, 2006; Patel et al., 2007). The GQs exhibit different molecularity and unimolecular GQ structure by default forms an intramolecular structure. For an intramolecular GQ to form at least four stretches of guanosines, containing a minimum of two contiguous G residues in each of the stretches are required. Typical gap between two consecutive G-stretches can range from one to seven nucleotides (Neidle and Balasubramanian, 2006). Since four G-stretches are required for an intramolecular GQ formation, typically it encompasses three sections that form the loops when the sequence adopts a GQ structure. The length and nucleotide composition of the loops play a crucial role in determining the stability of GQ structures (Risitano and Fox, 2004; Cevec and Plavec, 2005; Rachwal et al., 2007a,b,c; Guedin et al., 2010; Pandey et al., 2013). Moreover, the orientation of these strands also determines the topological classification of the GQ structure. A GQ is termed parallel if the polarities of all the strands are oriented in the same direction (Figure 3C) with respect to one another. In contrast, if each strand has an opposite polarity with respect to the two adjacent strands, the quadruplex is termed anti-parallel (Figure 3A; Neidle and Balasubramanian, 2006) However, possible mixed parallel-antiparallel strand orientations have also been observed (Esposito et al., 2007).

RNA GQs are more thermodynamically stable, compact structure compared to its DNA counterpart (Arora and Maiti, 2008, 2009; Zhang et al., 2011). It has been suggested that the 2′-OH of RNA may have stabilizing effects on RNA GQs, possibly through extended hydrogen bonding interactions with the phosphate oxygen backbone atoms, O4′ sugar oxygen and H-bond acceptors such as the N2 groups of quartet-forming guanines (Liu et al., 2002; Sacca et al., 2005; Collie et al., 2010). In addition, various independent studies have demonstrated the effect of variations in length and composition of loops on the stability of the GQ structure (Hazel et al., 2004, 2006; Risitano and Fox, 2004; Cevec and Plavec, 2005; Sacca et al., 2005; Guedin et al., 2008, 2010). Compared to DNA GQs, RNA GQs lacks structural heterogeneity and are almost exclusively known to adopt parallel conformation.

## Role of cations on G-quadruplex stability

The cation and quadruplex interaction is based primarily on ions which are located at the central channel formed due to the quartet arrangement. The positions of the ions can be along the quartet plane or between the planes of the quartet depending on the ion and the structure of the GQ. The ions contribute to charge screening, interaction with the loops and the grooves, thus playing an important role in GQ structure formation and their stability. Molecular dynamics simulation studies suggest that absence of coordinated cation at the center of the quartet destabilizes the GQ structure as it is electronically unfavorable (Špačková et al., 1999; Chowdhurdy and Bansal, 2001). Since Na^+^ and K^+^ are the physiologically relevant monovalent ions, the majority of the studies have examined their role and influence on GQ structure and stability. However, several other monovalent and divalent ions have also been shown to influence the structure and stability of GQs. The pattern that has emerged from the previously reported metal ion-GQ studies forms the basis of cation induced stability of GQ structures. These studies further elucidated the rationale for the stabilizing effects of cations on the GQ structure. Some of the very early studies on role of cations on the stability of GQ structures suggested the order as K^+^ > Ca^2+^ > Na^+^ > Mg^2+^ > Li^+^ and K^+^ > Rb^+^ > Cs^+^ (Hardin et al., 1992). Subsequently another study analyzed and reported interaction of GQ with type Ia and IIa cations and observed the following order of ions that stabilize GQs as Sr^2+^ > Ba^2+^ > Ca^2+^ > Mg^2+^ and K^+^ > Rb^+^ > Na^+^ > Li^+^ = Cs^+^ (Venczel and Sen, 1993). Although, there is a common notion that Li^+^ destabilizes GQ structure, we have repeatedly observed that it rather plays a neutral role wherein it neither stabilizes nor destabilizes GQ structures as has been observed by other research groups (Neidle and Balasubramanian, 2006).

Although, the physiologically relevant monovalent cations are the K^+^ and Na^+^, the list of cations which influences the GQ structure, thus far includes the monovalent cations Rb^+^, Cs^+^, ${\text{NH}}_{4}^{+}$, and Tl^+^ and divalent cations Sr^2+^, Ba^2+^, and Pb^2+^ that are known to promote GQ structures in some specific cases (Lee, 1990; Chen, 1992; Venczel and Sen, 1993; Nagesh and Chatterji, 1995; Basu et al., 2000; Smirnov and Shafer, 2000a; Miyoshi et al., 2001b; Cai et al., 2002). However, some divalent cations have been observed to disrupt GQ structures, for example, low concentrations of Mn^2+^, Co^2+^ or Ni^2+^ disrupt GQ structures even in the presence of K^+^ ions. When the GQ and monovalent cation concentrations are low enough, or the temperature is sufficiently high, divalent cations, for example, Ca^2+^, Co^2+^, Mn^2+^, Zn^2+^, Ni^2+^, and Mg^2+^ can induce destabilization of GQs (Blume et al., 1997; Hardin et al., 2001). Divalent cations were observed to destabilize the dimeric antiparallel GQ formed by d(G_4_T_4_G_4_)_2_ in the following order: Zn^2+^ > Co^2+^ > Mn^2+^ > Mg^2+^ > Ca^2+^ and induce a transition to a parallel GQ structure (Miyoshi et al., 2001b). Nevertheless, certain divalent metal ions stabilize GQs more than the others, but such effect depends on the structure of the GQ.

Several studies have reported an evaluation of the characteristics of divalent metal ions that affect the stability of the GQ. Marky and co-workers performed a series of biophysical studies using the thrombin aptamer in the presence of various metal ions and found out that Sr^2+^ and Ba^2+^ form thermodynamically more stable GQ than those formed by Mg^2+^ and Ca^2+^ due to their ionic radii (Kankia and Marky, 2001). Further, they characterized two major hydration contribution for the GQ folding; (i) dehydration of both cations and guanine O6 atomic groups (ii) the water uptake upon folding of a single strand into a GQ structure. Subsequently, an ESI-MS study on the same thrombin-binding aptamer by Vairamain et al. showed that the size of the metal ion is not the only factor that influences the stability of the GQ structure since they did not observe a smooth correlation between the ionic radii and the extent of adduct formation (Vairamani and Gross, 2003). Later on, Vairamain and co-workers performed another ESI-MS study on GQ formation by deoxyguanosine with various alkali earth metal ions (Sravani et al., 2011). The work provided evidences that the stability order of GQs formed with divalent cations as Sr^2+^ > Ba^2+^ > Pb^2+^ > Ca^2+^ >> Mg^2+^ suggesting the metal–oxygen bond is responsible for the stabilization of GQ structures. A computational approach toward the evaluation of the effect of interaction between alkaline earth metal cations and G-tetrads was conducted which found that the ability to stabilize follows the order Be^2+^ > Mg^2+^ > Ca^2+^ as the smaller ions are tightly bonded to the Gs suggesting the domination of electrostatic interaction in the cation–tetraplex systems (Deepa et al., 2011). A kinetic study of GQ formation in the presence of divalent cations was performed by the Liang group using an oligonucleotide sequence, d(GTG_3_TAG_3_CG_3_TTG_2_) which forms a unimolecular GQ in the presence of Pb^2+^ (Liu et al., 2012). They proposed that the Pb^2+^ induced GQ folding probably proceeds through a three-step pathway involving two intermediates.

Most of the divalent cation studies are limited to DNA G-quadruplexes rather than RNA GQs. The effect of divalent cation on the stability of RNA GQs was revealed in a recent study performed in our laboratory (Balaratnam and Basu, 2015). Using two distinct well-studied intramolecular GQs found in NRAS and MT3-MMP mRNAs, we identified the properties of metal ions that affect the GQ stability using a series of divalent metal ions. Our results demonstrated that the RNA GQs folded with K^+^ ions can be destabilized in the presence of divalent metal cations which can then be reversed by increasing the K^+^ ion concentration. The order of destabilization strength was observed to be Zn^2+^ > Cd^2+^ > Ni^2+^ > Co^2+^ >> Mn^2+^ > Mg^2+^ > Ca^2+^ > Sr^2+^ > Ba^2+^. The correlation of physicochemical properties of the divalent cations with the destabilization effect showed three major properties of the metal ions that determine its ability to stabilize an RNA GQ; (i) ionic radius (ii) hydration energy and (iii) binding strength toward the guanine O6. These findings are complementary to the properties that affect the stability of divalent metal cation induced DNA GQs that were discussed above.

Interestingly the effect of trivalent metal ions has been seldom studied. It was observed that trivalent ions destabilize GQ structures which were significantly reduced in the presence of metal chelators (Lu et al., 2015). Alternatively, another group using ESI-MS observed that the trivalent lanthanide metal ions (La^3+^, Eu^3+^, Tb^3+^, Dy^3+^, Tm^3+^) promoted stacking of the quartets (Kwan et al., 2007).

## Location of coordinating metal ions in G-quadruplexes:

### Monovalent cations

The negatively charged phosphate backbone of the nucleic acids provides the bulk of metal ion interaction sites which cause non-specific charge neutralization. However, the metal ions in the central channel of the GQ structure play the key role that directly contributes to the stability of GQs. The G-quartets stack upon each other to form GQ structures and the stacking also provides potential sites for cation coordination with four O6 atoms within the plane of a G-quartet or eight O6 atoms when between two stacked G-quartets. In fact, it has been established by *ab initio* calculations that the coordination of cation contributes more to the stabilization of GQ structure than the hydrogen bonding or stacking interactions (Gu et al., 1999).

The ionic radius (Table 1) is one of the major factors for the selection of cations for GQs. Ions such as K^+^ and ${\text{NH}}_{4}^{+}$ (ionic radii 1.33 and 1.48 Å respectively) are too large to be coordinated in the plane of a G-quartet, whereas Na^+^ (ionic radius 0.95 Å) is small enough to be coordinated within the plane of a G-quartet. Coordination interactions between guanine O6 atoms and cations contribute significantly to the overall stability of a GQ. For example, quantitative determination of ammonium peak intensity revealed that three ${\text{NH}}_{4}^{+}$ ions are placed among four quartets in d(G_4_T_4_G_4_) (Hud et al., 1999). In contrast, the smaller Na^+^ ion allows for in-plane coordination. Multiple Na^+^ ions are therefore not restricted to the spacing between G-quartets, and can move further away from each other to reduce electrostatic repulsions (Laughlan et al., 1994).

**Table 1:** **List of the effective ionic radii of the cations interacting with GQs Cotton and Wilkinson (1980)**.

**Ion**	**Coordination number**	**pm**
**MONOVALENT CATIONS**		
Li^+^	6	60
Na^+^	6	95
(NH4)^+^	6	148
K^+^	6	133
Tl^+^	6	140
Rb^+^	6	148
Cs^+^	6	169
**DIVALENT CATIONS**		
Co2^+^	6	74
Mn2^+^	6	80
Ni2^+^	6	69
Mg2^+^	6	65
Zn2^+^	6	74
Cd2^+^	6	97
Ca2^+^	6	99
Sr2^+^	6	113
Pb2^+^	6	121
Ba2^+^	6	135

Several other studies also defined the interaction of Na^+^ ions with GQs. A high-resolution crystal structure of the DNA hexamer d(TG_4_T), which has been refined to 0.95 Å suggests the formation of a tetramolecular parallel stranded GQ in the presence of Na^+^ ions. Interestingly, the two GQs with eight quartets are being stacked in a 5′ to 5′ orientation where seven Na^+^ ions are coordinated along the axial channel (Phillips et al., 1997). The Na^+^ ions in the terminal quartets are coplanar to the G-quartets. However, due to electrostatic repulsion between adjacent Na^+^ ions they are slightly displaced from the quartet plane and reside equidistant from the quartet planes with a slightly altered coordination geometry (Phillips et al., 1997). It was also observed that two distinct GQ structures are formed by d(TG_4_T) in the presence of Na^+^ and Tl^+^ ions. The Tl^+^ ions are coordinated between G-quartet planes as would be expected owing to its larger ionic radius (1.40 Å) (Caceres et al., 2004).

**Figure 4:**
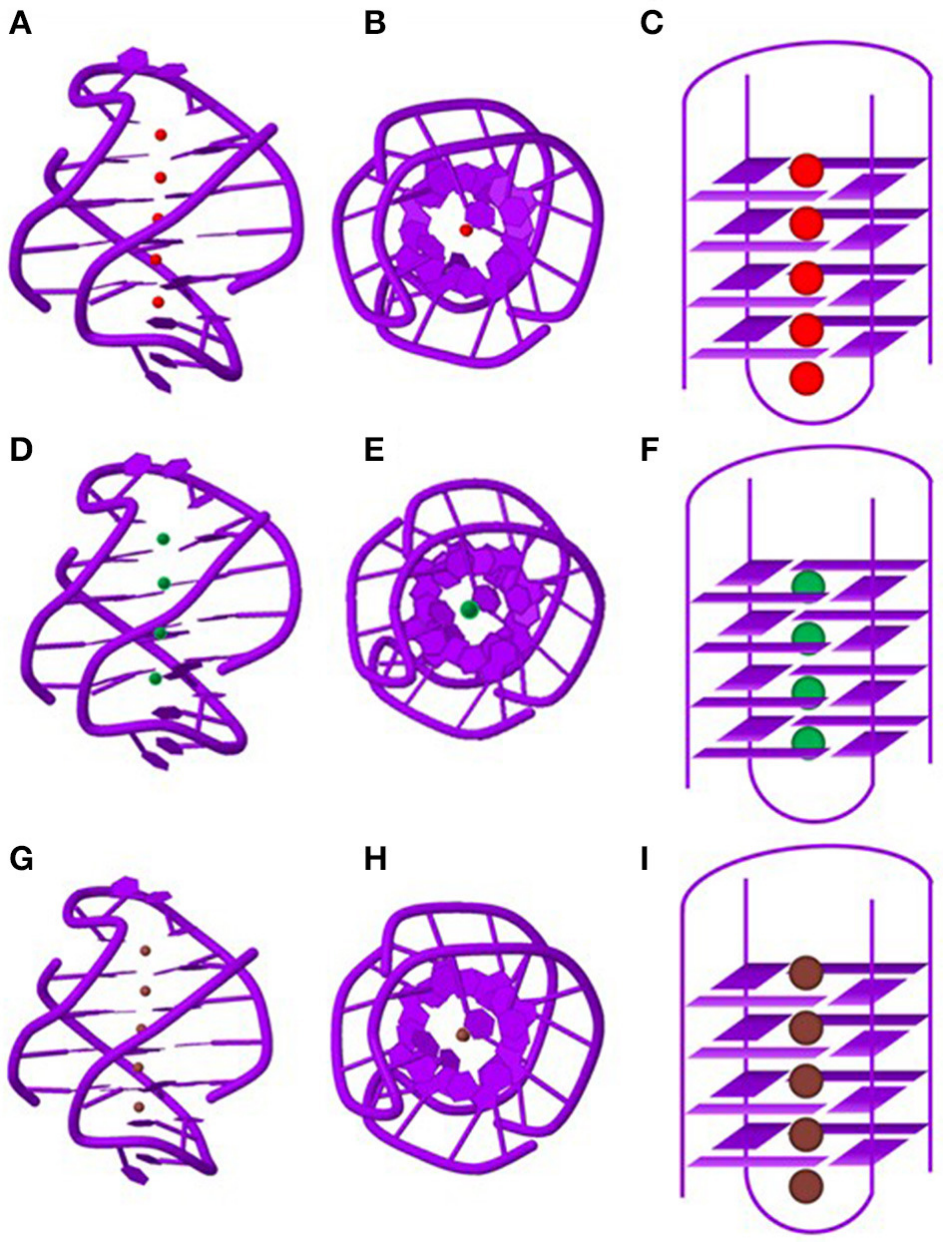
**Structure of the ***Oxytricha nova*** telomeric DNA d(GGGGTTTTGGGG) in the presence of different monovalent cations**. The side views of the crystal structure in the presence of K^+^ **(A)**, Na^+^ **(D)** and Tl^+^ **(G)** ions. A view shown down the central ion channel of the quadruplex in the presence of K^+^ **(B)**, Na^+^ **(E)** and Tl^+^ **(H)**. Schematic representations of the bi-molecular quadruplex showing positions of the monovalent cations in the central ion channel. K^+^ ions **(C)** and Tl^+^ **(I)** ions are sandwiched between G-quartets whereas Na^+^ **(F)** ions are located in plane with G-quartets. PDB entries (K^+^: IJPQ, Tl^+^: 2HBN and Na^+^: 1JB7).

Telomric G-rich sequences form various species are the most well characterized among GQs formed by naturally occurring sequences. Crystallographic study suggests that *Oxytricha nova* telomere sequence d(G_4_T_4_G_4_) forms a bimolecular quadruplex with four quartets. A linear row of five equidistant K^+^ ions lie along the central axis with an average distance of 3.38 Å between any two K^+^ ions in tandem (Figures 4A–C). The K^+^ ions are *ad libitum* equidistant from the planes of two adjacent G-quartets. Moreover, the outer K^+^ ions are located outside the GQ structure where they coordinate with the terminal quartets and O2 atoms of the loop residues, and water molecules (Haider et al., 2002). The same sequence in presence of Tl^+^, which is a perfect surrogate of K^+^ forms a structure which is very similar to the structure formed in presence of K^+^ (Figures 4G–I). Furthermore, the coordination of the Tl^+^ ions is similar to the K^+^ ions as described above (Gill et al., 2006). However, in presence of Na^+^ ions the structure indicates that the central Na^+^ ions are nearly coplanar with the G-quartets (Figures 4D–F). The outer Na^+^ ions are positioned beyond the planes of the flanking quartets more toward the loops where they coordinate with the quartet O6 and O2 atoms of the thymine bases in the loops (Horvath and Schultz, 2001; Haider et al., 2002).

In a 2.1 Å resolution crystal structure of the unimolecular GQ formed by d[AGGG(TTAGGG)_3_] in the presence of K^+^ ions (Parkinson et al., 2002), the K^+^ ions were found to be positioned equidistant from the stacked G-quartets, which is similar to the crystal structure described above. The structure of the human telomere repeat sequence d(TAGGGTTAGGGT) has been determined and similarly contains K^+^ ions that are coordinated between the adjacent G-quartets (Parkinson et al., 2002). On the other hand, the d(G_3_T_4_G_3_) forms a bimolecular structure containing three stacked G-quartets in presence of both Na^+^ and K^+^ (Smith et al., 1994; Keniry et al., 1995). NMR spectroscopy was used to monitor the competition of Na^+^ and K^+^ ions for coordination sites within the d(G_3_T_4_G_3_). Feigon group using NMR showed that two Na^+^ ions are displaced by two K^+^ ions within the GQ (Hud et al., 1996).

Similarly, the ability of Tl^+^ ions to compete with Na^+^ ions for coordination within the GQ [d(G_4_T_4_G_4_)]_2_ has been verified by solution-state ^1^H NMR. For the same GQ, the results from these experiments were similar to the K^+^–Na^+^ titration experiments discussed above (Feigon et al., 2001). A recent solution-state NMR structure of [d(G_4_T_4_G_4_)]_2_ folded in the presence of Tl^+^ ions has also confirmed that the Tl^+^ bound form of this GQ is similar to that of the K^+^ coordinated one indicating that Tl^+^ is an excellent surrogate for the K^+^ ion [need to mention the isotope and one old paper that describes its NMR property] (Gill et al., 2005). Molecular dynamics calculations have demonstrated similar localization of K^+^ ions between the neighboring G-quartets (Strahan et al., 1998). In addition to the alkali metal ions, the non-metallic monovalent cation ${\text{NH}}_{4}^{+}$ have also been shown to stabilize G-quartets to an extent that is similar to that observed for Na^+^ (Lee, 1990). This finding was later used to develop a probe for defining monovalent cation coordination sites in solution-state (Hud et al., 1998). Other NMR studies show that ${\text{NH}}_{4}^{+}$ ions within a GQ are positioned equidistant from stacked G-quartets probably due to its larger size (1.48 Å) (Hud et al., 1999).

Sodium ions can be coordinated within a G-quartet with three distinct ligand geometries: bipyramidal coordination sites directly between two G-quartet layers, octahedral coordination sites where the ion is coplanar with the G bases, and intermediate less symmetric positions. Sodium ions being less constrained by steric clashes than K^+^ ions can therefore, occupy a range of positions and in doing so reduce electrostatic repulsions between adjacent ions.

### Divalent cations

**Figure 5:**
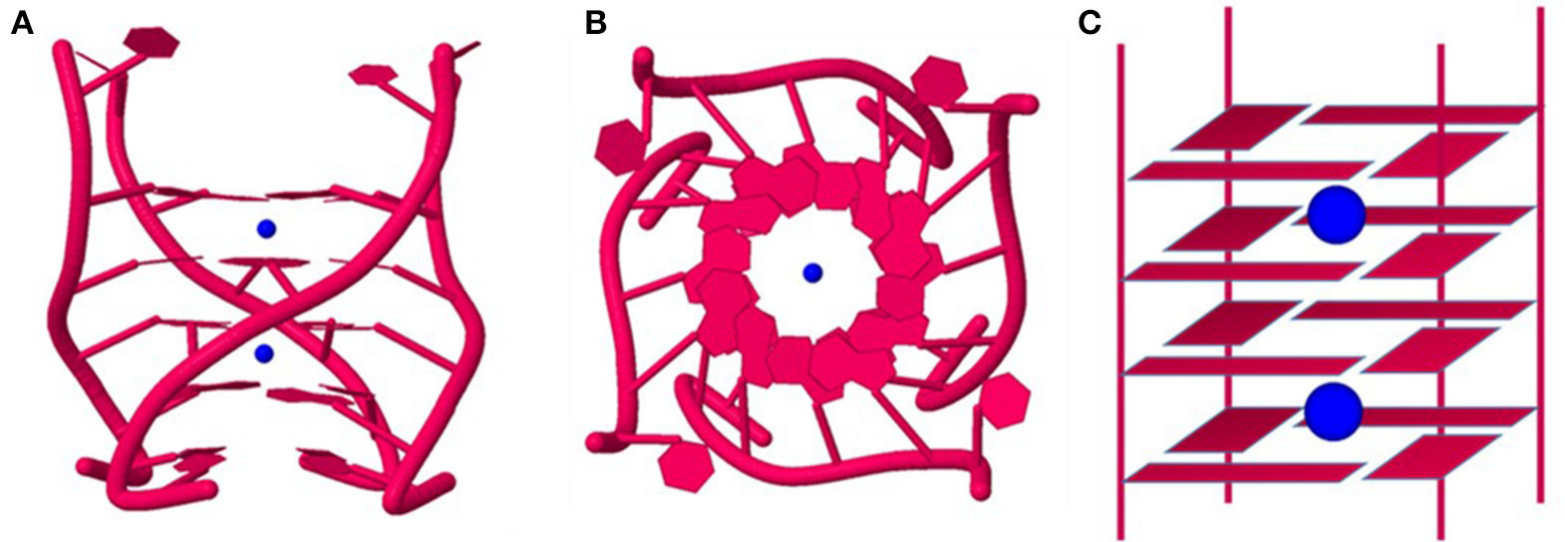
**Crystal structure of RNA G-quadruplex formed by the sequence (UGGGGU)4 in the presence of Sr^**2+**^ ions**. The side view **(A)** and the view down the central ion channel **(B)** of the quadruplex. The schematic representation **(C)** shows that each Sr^2+^ ion is sandwiched by two G-quartets. (PDB entry 1J8G).

Although, GQ folding is mostly studied as a monovalent cation aided process because of the physiological relevance, they can also be folded in the presence of a few of the divalent cations. The majority of the reported divalent cation-mediated GQ folding involved DNA GQs. Utilizing telomere-related sequences Chen showed that Sr^2+^ facilitates the intermolecular GQ formation (Chen, 1992). Although, two ions are of similar size, the authors argue that greater stabilizing effect of the divalent cation might be due to enhanced electrostatic interactions in the cavities as a result of increased charge density along with the enthalpy effect dominating over entropy effect. Interestingly, based upon a comparative study with monovalent cations, it was concluded that Sr^2+^-induced intermolecular GQs are thermally more stable than those induced by K^+^ (Chen, 1992). The ionic radius of Sr^2+^ is in between that of Na^+^ and K^+^ (Table 1); however, the energy from electrostatic repulsions between Sr^2+^ ions within a GQ could be four times that of monovalent cations. The crystal structure of the RNA GQ formed by the sequence (UGGGGU) in the presence of Sr^2+^ ions, suggests that the Sr^2+^ ions are coordinated between every other pair of stacked G-quartets and none of the cations are coordinated at the intervening positions (Figures 5A–C). The Sr^2+^ ions were found to be associated with eight carbonyl oxygen atoms of adjacent G-quartets. The electrostatic repulsion between Sr^2+^ ions resulted in vacant cation coordination sites within GQs (Deng et al., 2001).

Another divalent ion dependent folding GQ was discovered by Shafer and co-workers in thrombin binding aptamer which folds into an intramolecular GQ in the presence of Pb^2+^ (Smirnov and Shafer, 2000b). The precision and high affinity binding of thrombin aptamer with Pb^2+^ ions was later utilized to develop biosensor for the Pb^2+^ ion detection (Li et al., 2011; Jacobi et al., 2012; Brenneman et al., 2013; Zhang D. et al., 2014). Several attempts have been made to understand the structural details of divalent cation binding to GQs over the monovalent ion binding. Using a lipophilic guanosine analog, Davis and co-workers showed the complexation with Pb^2+^ to form G-quartets and G_8_ octamers both in solid-state and solution (Kotch et al., 2000). The NMR and crystal structures of these G_8_ complexes showed that the smaller and highly charged Pb^2+^ divalent ion templates a smaller G_8_ octamer cage than does K^+^. This tightly coordinated geometry between the Pb^2+^ and G-octamer explained why this complex is kinetically more stable than the G_8_-K^+^ ion complex. Subsequently, the same group by using X-ray crystallography and NMR spectroscopy showed that the anion in the pair with divalent cation can also significantly affect the kinetic stability of lipophilic GQs (Shi et al., 2001). Shafer group used extended X-ray absorption fine structure (EXAFS) measurements to characterize the Pb^2+^ ion binding to the thrombin binding aptamer (Smirnov et al., 2002). This study further provided evidence for binding of the Pb^2+^ ion in the area between the two quartets by coordinating to the eight surrounding guanine O6 atoms. Recently Xu group studied the effect of divalent cations on the solventless formation of G-quartet complexes with Au (III) (Zhang et al., 2015). They identified intra-quartet hydrogen bonding and ionic bonding as the driving force to form these complexes.

Although, the above-mentioned divalent cations bind to the cavity between the G-quartets of GQs, some other divalent cations bind into the grooves of the GQ structure. Marathias et al. using EPR and NMR determined the number and the location of the Mn^2+^ ion binding sites in thrombin aptamer GQ (Marathias et al., 1996). The NMR results and the predictions based on the electrostatic potentials showed two strong Mn^2+^ binding sites with one in each narrow groove of the aptamer structure. Niedle and co-workers reported a crystal structure of the intermolecular quadruplex in a mixed Ca^2+^ and Na^+^ ion environment (Lee et al., 2007). They observed an unexpectedly unequal distribution of Ca^2+^ ions in the GQ channel suggesting the Ca^2+^ and Na^+^ ions can readily interchange their positions. Thus, it can be argued that the divalent cations mainly show two modes of binding to GQ structures; (i) into the central cavity coordinated to the O6 atoms of Gs and (ii) into the grooves of GQs via interactions with the backbone of the DNA.

## Dynamics of metal ion binding and cation exchange in G-quadruplex structures

The GQ structures are generally associated with very slow off-rate. Given that the dynamicity of the bound metal ions are linked to the on and off rate of GQs, many studies were conducted to understand the phenomenon. The monovalent cations in the central channel in GQ structures were observed to be dynamic. The change in the localized ion concentration can change their coordination which can affect the stability of the GQ structure. The localized ion concentrations often vary and that may affect the structure and stability of GQs. It has also been established that often the stability of GQs directly correlates to their function. The modulation of GQ structure by a local change in ion concentrations might be a probable mechanism of regulation of function.

It was observed that the cations bound to the G-quartets exchange with ions in the bulk solution at a much higher rate (103 s^−1^) compared to the rate of the GQ opening which suggests that the GQ structure need not be disrupted to enable cation release, uptake or exchange (Hud et al., 1996). The residence lifetimes determined for Na^+^ ions tightly bound within DNA GQs are in distinct contrast to the kinetics of G-quartet base opening, which can be as slow as days to weeks (Smith and Feigon, 1993). NMR relaxation measurements suggest that lifetime of specifically bound Na^+^ ions to the sequence [d(G_4_T_4_G_4_)]_2_ is estimated to be 180 μs at 20°C (Deng and Braunlin, 1996). The Na^+^ ions coordinated within the tetramolecular GQ [d(T_2_G_4_T)]_4_ were shown to exchange rapidly compared to the bimolecular quadruplex [d(G_4_T_4_G_4_)]_2_. The difference in the structure of the dimeric and tetrameric GQs, especially the role of the loops was implicated in the cation exchange. On the other hand, the ${\text{NH}}_{4}^{+}$ ion has a longer binding lifetime (>250 ms), probably due to a larger ionic radius. It has been suggested that G-quartets might alter or partially denature to allow for the movement of K^+^ or ${\text{NH}}_{4}^{+}$ ions in their coordination states, whereas sodium ions may move freely along the central axis owing to its much smaller ionic radius (Hud et al., 1999; Chowdhurdy and Bansal, 2001; van Mourik and Dingley, 2005).

The Plavec group significantly contributed to our understanding of the dynamics of ion movements in the central channel of the GQ. They studied the movement of ${\text{NH}}_{4}^{+}$ ions in different GQs using NMR and provided insight into the dynamics of the ions. Evidence that different ions can simultaneously coordinate with GQ structures was found when K^+^ ions were titrated into the Na^+^ ions containing dimeric GQ structure formed by G_3_T_4_G_3_. The Na^+^ ions were observed to replace the ${\text{NH}}_{4}^{+}$ ions in the GQ formed by d(G_4_T_4_G_4_) when the Na^+^ concentration was increased. A mixed ion coordination was observed when K^+^ was titrated into a solution of GQ formed by [d(G_3_T_4_G_4_)]_2_ in the presence of ${\text{NH}}_{4}^{+}$ ions. The ${\text{NH}}_{4}^{+}$ ions were similarly found to replace Na^+^ ions inside the GQ. The preference for ${\text{NH}}_{4}^{+}$ over Na^+^ ions for the two internal coordination sites is considerably less than the preference for K^+^ over ${\text{NH}}_{4}^{+}$ ions. The cation binding sites displayed differential affinity during the cation exchange where exchange at one site always preceded the other (Hud et al., 1996; Šket et al., 2004, 2005). Furthermore, it was observed that in the same GQ structure at 25°C there was no ${\text{NH}}_{4}^{+}$ ion movement although there was very slow movement at 35°C of ${\text{NH}}_{4}^{+}$ ions to the bulk solution from the outer binding site with residence lifetime of 1.2 s. The restricted movement of ${\text{NH}}_{4}^{+}$ ions was ascribed to steric hindrance of the loops and the rigidity of the GQ (Šket and Plavec, 2007; Podbevšek et al., 2008). The group also observed that d[G_4_(T_4_G_4_)_3_] forms identical unimolecular GQ in the presence of both Na^+^ and ${\text{NH}}_{4}^{+}$ ions where four quartets harbor three ion-binding sites and that the ${\text{NH}}_{4}^{+}$ ions had no unidirectional ion movement, instead moved back and forth through the central cavity of the GQ to exchange with ions in bulk solution. In this case, the rigidity of the quartets and steric hindrance of the loops contribute to the 5-fold difference in the exchange rate through the outer G-quartets. They concluded that the ion movement depends on several factors such as thermodynamics at the individual binding sites, steric hindrances, and constraints imparted by the quartets and the loops (Podbevsek et al., 2007).

The same group used the 15-mer oligonucleotide d[G_2_T_2_G_2_TGTG_2_T_2_G_2_] more commonly known as the thrombin binding aptamer (TBA) which forms an antiparallel unimolecular GQ in the presence of ${\text{NH}}_{4}^{+}$ ions. These ions exchange between the inner binding site and bulk solution at 15°C with exchange rate constant of 1.0 s^−1^. A non-canonical T:T base pairing significantly affects access of bulk ions into the cation binding site within the GQ core. Moreover, two Gs in a quartet bend toward the two loops and away from the bound ${\text{NH}}_{4}^{+}$ ions thereby exposing them for more efficient exchange with the bulk ions and water (Trajkovski et al., 2009). Furthermore, it was observed that the ${\text{NH}}_{4}^{+}$ ions have been shown to move faster between the interior of the tetramolecular structures and the bulk solution in comparison with the unimolecular and bimolecular GQs (Šket and Plavec, 2010). Similarly, they observed that orientation of the strands in the GQ and the base orientation also influence the ion exchange in GQ structures. The rate constants for ${\text{NH}}_{4}^{+}$ ion movements was observed to be slower at the 5′ -end and through an all-*syn* G-quartet compared to 3′-end and all-*anti* quartet respectively (Šket et al., 2012).

To study the dynamics of a monovalent cation, surrogate ions whose physicochemical properties are closer to K^+^ can be used. For example, the Tl^+^ cation was used as a probe to study the d(T_2_G_4_T)_4_ by NMR. The Tl^+^ ions exchange with unbound ions in solution freely with an estimated residence lifetime of 3 μs within the GQ (Basu et al., 2000). The exchange of ions in a GQ structure is therefore strongly dependent on the bulk of the ions, molecularity and the structure of the GQ.

## Cation-induced polymorphism of G-quadruplex structures

### G-quadruplex polymorphism in presence of monovalent cations

The polymorphism of GQ structures is determined by strand orientation (i.e., parallel vs. antiparallel), the conformation of the glycosidic bonds, and the loop topology (e.g., lateral loops vs. diagonal loops). These variations in the structure might be due to several reasons such as the cationic coordination, π-stacking interactions, hydrogen bonding and hydrophobic effects. The GQ structure formed by d[AGGG(TTAGGG)_3_] exhibited conformational changes in the glycosidic bonds in the presence of K^+^ and Na^+^ as was observed by both NMR and CD experiments (Balagurumoorthy and Brahmachari, 1994). In the presence of Na^+^, the GQ comprises of three quartets with *syn*–*anti*–*anti*–*syn* conformations in each G-quartet (Gilbert and Feigon, 1999). The crystal structure of the GQ formed by the same sequence in the presence of K^+^ showed that the strands were parallel and all G residues adopted *anti* conformation. Interestingly the overall structure in the presence of K^+^ was an asymmetric propeller shape, alternatively, the structure was globular in the presence of Na^+^ ions (Parkinson et al., 2002). G-quadruplexes formed by oligonucleotides with a sequence motif dG_4_-loop-dG_4_, where the loop consisted of one of the following 1′,2′-dideoxyribose, propanediol, hexaethylene glycol, and thymine residues, showed the formation of a single structure in the presence of Na^+^ ions, whereas multiple structures were observed in presence of K^+^ or ^15^${\text{NH}}_{4}^{+}$ ions (Cevec and Plavec, 2005). Similarly, a single GQ structure of [d(G_4_T_4_G_3_)]_2_ was observed in the presence of Na^+^ ions, while several structures were observed in the presence of K^+^ or ^15^${\text{NH}}_{4}^{+}$ ions suggesting an influence of cation coordination on the polymorphism of GQ structures (Črnugelj et al., 2002). One of the conformations adopted by GQs is the chair form and K^+^ ions are known to stabilize the “chair” type structures with lateral loops, however, these structures cannot form in the absence of K^+^ ions (Marathias and Bolton, 1999). The thrombin binding aptamer is an example of a chair-type GQ that forms in the presence of K^+^ ions (Marathias and Bolton, 2000).

Fragile X syndrome triplet repeat sequence d(TG_2_CG_2_C) provides a unique example of metal ion dependent polymorphism. It forms a tetramolecular parallel GQ in the presence of K^+^ ions at neutral pH. Interestingly, under similar conditions when the K^+^ is replaced by Na^+^ ions it forms an antiparallel GQ (Patel et al., 2000). In contrast, the analogous oligonucleotide d(TG_3_CG_2_C) forms a tetramolecular GQ in solutions containing either Na^+^ or K^+^ (Patel et al., 2000). Another interesting discovery involves the telomeric DNA repeats of *Tetrahymena* d(T_2_G_4_)_4_ that formed a unimolecular structure in sodium phosphate buffer while adopting a multi-strand GQ structure in potassium phosphate buffer (Hardin et al., 1991). Studies on oligonucleotides derived from the *Oxytricha nova* telomere repeat, d(T_4_G_4_)_2_ and dT_6_(T_4_G_4_)_2_ showed that in the presence of 10–50 mM NaCl, they form simpler, hairpin structures stabilized by GC base pairs rather than G-quartets. However, in the presence of 150 mM KCl, these sequences are converted to parallel, tetramolecular GQs (Laporte and Thomas, 1998). On the other hand, the human telomeric double repeat sequence d(TTAGGG)_2_ at lower Na^+^ ion concentration remained as a single-strand, however, at higher Na^+^ concentration transformed into parallel and antiparallel GQ structures (Rujan et al., 2005).

**Figure 6:**
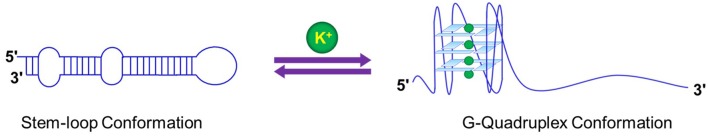
**Structural switch in response to ionic environment in miRNA 92b may influence the processing and the biogenesis of the G-quadruplex harboring miRNAs**.

Switching or co-existence of hairpin and GQ structures has also been observed with RNA sequences. A designed RNA sequence which could either form a GQ structure or a hairpin but not simultaneously, formed GQ structure in presence of K^+^ ions, but in presence of Mg^2+^ converted to the hairpin structure (Bugaut et al., 2012). In a separate study our group showed that microRNA maturation from pre-microRNA was modulated by the equilibrium of the hairpin to GQ switch (Figure 6). Since the hairpin is an essential structural requirement for maturation, the population of the two structural variants in equilibrium dictates the amount of matured miRNA (Mirihana Arachchilage et al., 2015; Pandey et al., 2015). A very similar hairpin to GQ transition was observed in the DNA GQ forming sequence in WNT1 promoter. The hairpin transition to GQ was observed to be quite slow at 4800 s indicating that such switching may be a slow process (Kuo et al., 2015).

### Monovalent cation induced change in the molecularity of G-quadruplexes

The oligonucleotide with a single repeat of the human telomere sequence, d(TTAGGG), forms a tetramolecular parallel-stranded GQ at low K^+^ concentrations, and the structure stacked and aggregated at higher concentrations of K^+^ ions (Kato et al., 2005). Another interesting case is that of the oligonucleotide d(G_2_AG_2_AG) which forms a bimolecular GQ at low Na^+^ concentration while a tetramolecular quadruplex at higher 150 mM Na^+^ (Kettani et al., 2000). The four-stranded dimeric quadruplex comprises a unique hexad motif as opposed to a two-stranded “arrowhead” motif (Kettani et al., 1999). Furthermore, d(G_3_T_4_G_3_) and d(G_4_T_4_G_4_) form only bimolecular GQ structures in the presence of Na^+^ ions while in presence of K^+^ ions both bimolecular and tetramolecular GQ structures were observed (Balagurumoorthy et al., 1992; Strahan et al., 1998).

### G-quadruplex polymorphism in the presence of divalent cations

Although, the structural polymorphism of GQs was first discovered with monovalent cations, later studies discovered that it can also be true in the case of divalent cations. Sen and co-workers showed the divalent ion dependent structural polymorphism of a GQ that was derived from the *Saccharomyces* telomeric consensus sequence (Venczel and Sen, 1993). They discovered that dramatic switches in the formation of parallel vs. antiparallel structures occurred in the presence of Mg^2+^ and Ba^2+^. Thermodynamic studies on *Oxytricha* telomere DNA d(G_4_T_4_G_4_) in the presence of various divalent ions also showed a conformational switch of GQ from antiparallel to parallel (Miyoshi et al., 2001a,b). It was found that divalent cations destabilize the antiparallel conformation and at a higher concentration of a divalent cation they can induce a transition from an antiparallel to a parallel GQ conformation. (Miyoshi et al., 2001b) Further studies performed on the d(G_4_T_4_G_4_) GQ showed that Ca^2+^ ions induce a transition from the antiparallel to parallel GQ and finally adopting the G-wire structure. The quantitative parameters showed that at least two Ca^2+^ ions are required for the transition, which undergoes through multiple steps involving the Ca^2+^ binding, isomerization, and oligomerization of d(G_4_T_4_G_4_). (Miyoshi et al., 2001a) Additionally, Majhi et al used a GQ adopted by the DNA oligomer d(GTG_3_TAG_3_CG_3_T_2_G_2_) to decipher the metal ion-dependent structural polymorphism in the presence of K^+^ and Pb^2+^ ions. (Majhi and Shafer, 2006) Although, the K^+^ ions induce both unimolecular and multi-stranded structures, Pb^2+^ ions resulted only in an antiparallel unimolecular structure. Amazingly, a single divalent metal ion (Pb^2+^) suffices to fully form this antiparallel GQ. Collectively, these studies provide evidence for structural polymorphisms of GQs induced by divalent cations.

## Rationale for the selectivity of cations that coordinate with GQ structures

The size of a cation and its energy of hydration both contribute to cation selectivity and the stability of a GQ structure. Generally, the hydration energies of monovalent cations are inversely proportional to their ionic radii. The energy required for K^+^ coordination within a GQ is less than that required to dehydrate Na^+^. It has been suggested that the preference of potassium for the stabilization of GQ structures over Na^+^ ions is a result of two separate events combined together. Although, it has been suggested that the free energy of Na^+^ binding to a quadruplex is more favorable than that of K^+^, the cost of energy for the dehydration of the Na^+^ ion far exceeds that for K^+^ ions. The combination of the two factors results in a free energy change in favor of the K^+^ ions (Hud et al., 1996; Gu and Leszczynski, 2002). The net difference between the free energy of coordination within a GQ and the free energy of dehydration ultimately determine cation selectivity by the GQs (Ross and Hardin, 1994; Hud et al., 1996; Sacca et al., 2005). Quantitative analyses demonstrated that the preferred coordination of K^+^ over Na^+^ is in fact driven by the greater energetic cost of Na^+^ dehydration with respect to K^+^ dehydration, whereas the intrinsic free energy of Na^+^ coordination within [d(G_3_T_4_G_3_)]_2_ is actually more favorable than K^+^ coordination. Overall, the conversion of the G-quadruplex [d(G_3_T_4_G_3_)]_2_ from its Na^+^ form to the K^+^ form is associated with a net free energy change of −1.7 kcal mol^−1^ (Hud et al., 1996). Subsequent calculations have provided additional support for the argument that cation dehydration is the dominant free energy that determines Na^+^ vs. K^+^ selectivity by GQs (Gu and Leszczynski, 2002).

## Divalent cation based G-quadruplex ligands

Various ligands have been developed and reported to bind to GQs and modulate their regulatory roles in biological processes. While some of these ligands facilitate the folding and/or stabilize the GQs others can destabilize or unfold them. Different strategies have been utilized to increase the binding of these ligands to GQs. Two of the most commonly utilized strategies are, (i) π-π stacking interactions and (ii) binding to grooves or backbone of GQs. Recent studies deployed the divalent metal ions for designing of the GQ binding ligands with enhanced affinity and selectivity. Suntharalingam et al. tested the binding of Cu^2+^, Pt^2+^, and Zn^2+^ complexes with terpyridine based ligands to human telomeric (HTelo) and c-myc GQ sequences (Suntharalingam et al., 2010). The dicopper and diplatinum complexes showed very strong bindings to GQs over duplex DNA. They proposed that these complexes bind to GQ via a combination of π-π end-stacking interaction and electrostatic/metal-phosphate interactions. Yatsunyk and co-workers used TMPyP4 (5,10,15,20-tetra(N-methyl-4-pyridyl)porphyrin) which is a well-studied GQ binding ligand incorporating Zn^2+^, Pt^2+^, and Cu^2+^ as the central ion to investigate the folding of a bimolecular GQ (Bhattacharjee et al., 2011). They found that ZnTMPyP4 is capable of inducing the GQ formation, speeding up the folding and stabilizing GQ than other divalent cation-TMPYP4 complexes. This unique ability of ZnTMPYP4 might be due to the axial water molecule which is absent in other complexes. Sissi Lab used Ni^2+^ ions to increase the selectivity of phenanthroline complexes toward GQ (Musetti et al., 2013). They proposed that the extended planar surface achieved through metal coordination promotes stacking onto a guanine quartet which caused the enhanced selectivity. Another study using a Cobalt (III) porphyrin bearing two water molecules as axial ligands on the central metal ion showed that it selectively and strongly binds to the 5′ end G-quartet of the GQ (Sabater et al., 2015). Collectively, the divalent cations can be used to design GQ binding ligands with enhanced selectivity and specificity.

## Conclusion

Metal cations have an intimate and intrinsic association with GQs of all hues acting as a key stabilizer of such structures. Dehydration energy, ionic radius, and binding strength toward the guanine O6 appear to be the key set of factors determining the choice of metal ion binding to a GQ. A major number of coordinations of the metal ion in the central cavity of a GQ are with the carbonyl oxygens of the guanines. A specific G-rich sequence can adopt different GQ structures based upon the metal ion, on the other hand, the same metal ion can have different effects based on the nucleotide composition of the G-rich sequence. Metal ion-based complexes can provide a novel class of small molecule ligands for manipulating GQ function which can derive therapeutic values.

Overall we have attempted to comprehensively review certain aspects of metal ion-GQ interactions and in case we left out any work that should have fallen under the purview of this review it is absolutely inadvertent. In any case our apologies for inadvertently leaving out any relevant work.

## Author contributions

All authors listed, have made substantial, direct and intellectual contribution to the work, and approved it for publication.

## Funding

Part of the research described in the review was funded by NIH grant (1R15GM116110-01) to SB. GM was partially supported by GSS, KSU.

### Conflict of interest statement

The authors declare that the research was conducted in the absence of any commercial or financial relationships that could be construed as a potential conflict of interest.
